# Does the tutors' academic background influence the learning objectives in problem-based learning?

**DOI:** 10.3205/zma001301

**Published:** 2020-02-17

**Authors:** Matthaeus C. Grasl, Karl Kremser, Jan Breckwoldt, Andreas Gleiss

**Affiliations:** 1 Medical University of Vienna, Department of Otorhinolaryngology, Vienna, Austria; 2 Medical University of Vienna, Teaching Center, Vienna, Austria; 3 University of Zurich, Medical Faculty, Institute of Anesthesiology, Zurich, Switzerland; 4 Medical University of Vienna, Center for Medical Statistics, Informatics, and Intelligent Systems, Section for Clinical Biometrics, Vienna, Austria

**Keywords:** problem-based learning, tutors` academic backgrounds, near-peer student tutors, intended learning objectives, additional learning objectives

## Abstract

**Background: **Problem-based learning (PBL) is an essential element of the curriculum of the Medical University of Vienna (MUV) and is performed in an eight steps model with: clarifying, defining, analysing, shifting & sorting, identifying learning objectives, going to learn and coming back to talk and feedback. With an annual intake of up to 740 students the MUV has to recruit PBL tutors from various academic backgrounds including undergraduate near-peer students. Therefore, we were interested to see whether a tutor's academic background had an influence on the resulting PBL sessions as reflected by the percentage of learning objectives (LOs) which were actually achieved in relation to the intended LOs.

**Methods: **For each PBL session “intended learning objectives” (ILOs) were defined. ILOs were communicated to all tutors by means of PBL session guides in order to provide homogenous learning opportunities to all students. However, it was not mandatory to reach all ILOs. The PBL coordination regarded a range of two thirds to three quarters of ILOs as a desirable goal. For analysis we retrieved data concerning ILOs, characteristics of tutors and PBL groups from the institution's PBL quality assurance system.

**Results:** From 2012-2014, 216 PBL groups were facilitated by 106 tutors with different academic backgrounds. On average, 70.8% (95% CI: 69.2-72.5%) of the ILOs were achieved; MUV clinicians reached 74.3% (70.8-77.8%), MUV non-clinicians 74.2% (71.7-76.6%), external faculty (clinicians and non-clinicians) 68.6% (64.4-72.8%), and near-peer students 64.7% (61.8-67.7%). Statistically significant differences were found between near-peer students and MUV clinicians (p<.001) as well as MUV non-clinicians (p<.001).

**Conclusions: **ILOs were reached within a satisfactory range. However, groups taught by near-peer students reached significantly fewer ILOs than groups taught by MUV faculty tutors. This finding raises the question whether tutor training for near-peer students should be intensified. Also, further research is needed to explore the group dynamics of student-led PBL groups.

## Zusammenfassung

**Zielsetzung: **Problemorientiertes Lernen (POL) ist ein wesentlicher Bestandteil des Lehrplans an der Medizinischen Universität Wien (MUW) und wird in einem achtstufigen Prozess durchgeführt: Klärung unbekannter Begriffe, Definition des Problems, Sammlung von Hypothesen und Ideen, Ordnung und Bewertung von Hypothesen, Lernzielformulierung, Lernzeit, Synthesen und Feedback. Die MUW nimmt jährlich bis zu 740 Studierende auf und muss daher POL-Lehrende mit verschiedenen akademischen Hintergründen, darunter auch Studierende aus höheren Semestern, beschäftigen. Daher waren wir interessiert zu sehen, ob der akademische Hintergrund von POL-Lehrenden einen Einfluss auf die resultierenden POL-Veranstaltungen hat, und sich im Prozentsatz der tatsächlich erreichten Lernziele im Verhältnis zu den gewünschten Lernzielen wiederspiegelt.

**Methoden: **Für jede POL-Veranstaltung wurden „gewünschte Lernziele“ bestimmt. Diese wurden allen Lehrenden in einer POL-Schulung mitgeteilt um allen Studierenden einheitliche Lernangebote bereitzustellen. Allerdings war es nicht verpflichtend alle gewünschten Lernziele zu erreichen. Die POL-Koordination erachtete eine Bandbreite von zwei Drittel bis drei Viertel von gewünschten Lernzielen als erstrebenswertes Ziel. Für die Analyse haben wir Daten zu den erwünschten Lernzielen, den Merkmalen der Lehrenden und POL-Gruppen aus dem POL-Qualitätssicherungssystem der Institution abgefragt.

**Ergebnisse: **Von 2012 bis 2014 wurden 216 POL Gruppen von 106 Lehrenden mit unterschiedlichen akademischen Hintergründen betreut. Im Durchschnitt wurden 70.8% (95% KI: 69.2-72.5%) der gewünschten Lernziele erreicht. MUW Kliniker erzielten 74.3% (KI: 70.8-77.8%), MUW Nicht-Kliniker 74.2% (KI: 71.7-76.6%), externe Lehrende (Kliniker und Nicht-Kliniker) 68.6% (KI: 64.4-72.8%), und Studierende 64.7% (KI: 61.8-67.7%). Es wurden statistisch signifikante Unterschiede zwischen Studierenden und MUW-Klinikern (p<.001) sowie MUW-Nichtklinikern (p<.001) gefunden.

**Schlussfolgerung: **Die erwünschten Lernziele wurden in einem zufriedenstellenden Bereich erreicht. Gruppen von Studierenden die von nahezu gleichrangigen Studierenden moderiert wurden, erreichten jedoch signifikant weniger gewünschte Lernziele als Gruppen von Lehrkräften der MUW. Das wirft die Frage auf, ob das Moderationstraining für Studierende intensiviert werden sollte. Darüber hinaus sind weitere Forschungsarbeiten erforderlich, um die Gruppendynamik von studentisch angeführten POL-Gruppen zu untersuchen.

## 1. Introduction

### 1.1. Background

Problem-based learning (PBL) is a widely applied method for learning and teaching, with great emphasis on collaborative learning in a functional context [1], [2], [3], [4], [5], [6], [7], [8], [9], [10], [11], [12], [13], [14], [15], [16]. PBL is believed to provide students with competencies suitable for problem solving [11], [17] which may also be applied to a clinical context [18], [19]. The educational outcome of PBL is determined by the quality of group interaction, the students’ motivation to learn, the quality of PBL cases [20], [21], [22] and tutors` qualification [23], [24]. The tutors take the responsibility for the quality of this particular type of learning designed to stimulate students to further investigate problems, especially where cognitive conflicts arise. The role of the tutor is to structure the students` learning process, to encourage them to draft concepts, interlink knowledge and to stimulate interaction between students by asking for clarifications and by formulating goal-oriented questions [3], [25], [26], [27], [28]. The tutor should be able to recognize the gaps between knowledge of the students and intended learning objectives. Their major tutor's role is to activate prior knowledge which helps the students solve the case at hand [29]. 

#### 1.2. Problem

Large medical schools, such as the Medical University of Vienna (MUV) with a total annual intake of up to 740 students, need a large number of tutors. Such a large number of students cannot be managed by academic teachers alone, calling for an alternative solution. We therefore introduced faculty from all medical specialties, basic sciences (non-clinical disciplines), faculty from outside the MUV and near-peer students who were further advanced in their studies [30]. In this situation, concern had been raised that the different tutors' academic backgrounds might influence PBL facilitation, a problem which would also apply to other large medical schools. 

#### 1. 3. State of Research 

The influence of tutor qualification on the quality of learning, the learning process and learning outcomes has been analysed by several authors [31], [32], [33], [34]. Results differ and depend on the availability of PBL within the curriculum and on other local factors. Regehr et al [35] did not find any differences between the outcome measures for students led by content-expert and nonexpert tutors. In addition, Burgess et al. [36] describe in their systematic review a variety of learning benefits for student tutors. 

Finally, none of the studies has analysed/compared tutors of several different academic backgrounds at a medical school of the size of the MUV.

#### 1.4. Aim

We were particularly interested to see if all tutors would be able to sufficiently guide the PBL process. As the primary outcome measure of this study we defined the percentage of “intended learning objectives” (ILOs) achieved by the PBL groups. ILOs were the LOs predefined for each PBL case by the MUV's PBL steering group (PBL coordinator and module coordinator). ILOs were broadly defined (by content and by LO dimensions, such as knowledge, skills, attitudes). LOs were provided to the tutors for more efficient guidance of the group process. As the secondary line of enquiry, additional LOs outside of the predefined spectrum were quantified and classified by thematic analysis. 

The final purpose of the study was to identify whether tutor groups significantly differed in respect to the proportion of ILOs and therefore might need additional training (learning format, content of cases, content of curriculum) [37].

## 2. Methods

### 2.1. General conditions and sample 

We conducted this study in the context of the PBL curriculum at the Medical University in Vienna, Austria (MUV). In the MUV curriculum, PBL was included in the first semester with four cases, and in the second and third semester with six cases each. Attendance at the whole series was mandatory for all students. 

During the study period 106 different tutors were involved (22 MUV clinicians, 33 MUV non-clinicians, 13 external faculty (clinicians and non-clinicians), 38 near-peer students). They were randomly allocated to seminar groups.

All tutors and all student groups of 9-11 students worked on each case for two face-to-face sessions of 90 minutes. Between the face-to-face sessions (step 6 of PBL, according to an 8-step PBL format) the amount of self-regulated learning time varied individually. In the first session, the group undertook structured problem analysis and agreed on LOs for self-study, the results of which were discussed in the reporting phase during the second session. All students were instructed to work on the same LOs during their self-studies (not splitting up their learning into fragmented “team work”). While students were not informed about the ILOs of the case, PBL tutors were provided with a written guide giving the ILOs and theoretical background for each case. The students involved in this study were in their third semester of studies and thus already familiar with PBL. The tutors came from clinical and pre-clinical institutions of the MUV, from external institutions (clinicians and pre-clinicians combined), or were near-peer students who had at minimum entered the third year of medical studies. All tutors had undergone a two-day course introducing the principles of PBL within the MUV curriculum, including practical training sessions in small groups. In addition, tailored seminars of 2-3 hours were held for all tutors for the specific cases of a semester in order to ensure homogeneity of LOs derived from the case [38], [39]. Immediately after the first session of each case the students placed the LOs in the e-learning platform of the MUV (Moodle).

Participation in the case-specific seminars was voluntary in the academic year 2012, while in 2013 and 2014 it was made mandatory – with the aim that all PBL groups should arrive at comparable LOs, especially in regard to the end-of-year assessment. However, based on the self-regulatory nature of PBL at the MUV this was not an absolute obligation. PBL groups were free to select their individual LOs specific to the learning progress of the group. The expected study time, depending on students' knowledge, was supposed to range between two and four hours. 

The curriculum committee proposed a proportion of two thirds to three quarters of the ILOs as the desirable range because PBL should support the learning goals of the parallel curricular modules sufficiently.

We collected tutors and students' data from all PBL groups of the third semester in the academic years 2012-2014. Each years' cohort consisted of 72 PBL groups, resulting in a total of 216. We included the academic year 2012 to be able to control for a potential influence of voluntary vs. mandatory participation at the case specific seminars. We used the written routine session reports of LOs worked out by the PBL groups which were routinely collected by students or tutors for feedback and quality control by the PBL programme director. In total, the six cases included in the analysis contained 34 ILOs (6, 7, 7, 6, 4 and 4). Matching was performed by two raters, one clinician and one basic scientist. Less than 1% of the matches differed and were discussed by the raters to find accordance. If the LOs determined by the students matched the ILOs, we classified them as “achieved”. Other LOs were classified as “additional”. We registered additional LOs outside the ILOs to find out if a case needs to be modified. The researchers involved in this analysis were blinded with regard to tutor and PBL group characteristics. All data were transferred to an electronic database using SPSS, version 19.0 and analysed with SAS, version 9.4. 

#### 2.2. Sample size calculation 

Based on the experiences of previous years (2010-2011), we expected a proportion of achieved ILOs of 60-75% in each PBL group, assuming a normal distribution across PBL groups. Given that data of 216 PBL groups would be available from three years' cohorts, an estimated standard deviation of 10 (or 12) percentage points was calculated to result in 95% confidence intervals with a width of 2.6 (or 3.2) percentage points. We regarded this a sufficient accuracy for the primary research question which would also allow sufficient statistical power for group comparisons of the secondary research question. 

#### 2.3. Statistical analysis 

For each PBL group, the proportion of achieved ILOs among the total number of 34 ILOs defined for all six cases of the semester was calculated. This proportion was reported by mean, standard deviation (SD) and 95% confidence interval (CI) and graphically depicted by academic year and tutor's academic background using box plots. The distribution of groups among semesters and tutor’s academic background was given by counts and percentages and tested for independence using Chi-square test. The dependence of the proportion of achieved ILOs on academic year semester and tutor’s academic background was investigated using two-way ANOVA. The interaction of semester and tutor’s academic background was removed from the model due to non-significance. Least square means and 95% CI's were reported based on this model. Pairwise post-hoc comparisons were corrected for multiple testing using Tukey’s method. The potential gender difference of the outcome variable was investigated by t-tests. The secondary outcome, proportion of additional LOs, was investigated using Kruskal-Wallis tests due to non-normal distributions. All calculations have been performed using SAS 9.4. Two-sided p-values ≤ 0.05 were regarded as statistically significant. As a “clinically relevant” difference, we regarded a deviation by more than 10% in relative frequency of ILOs.

## 3. Results

### 3.1. Proportion of ILOs achieved in respect to years' cohorts 

**Figure 1:**
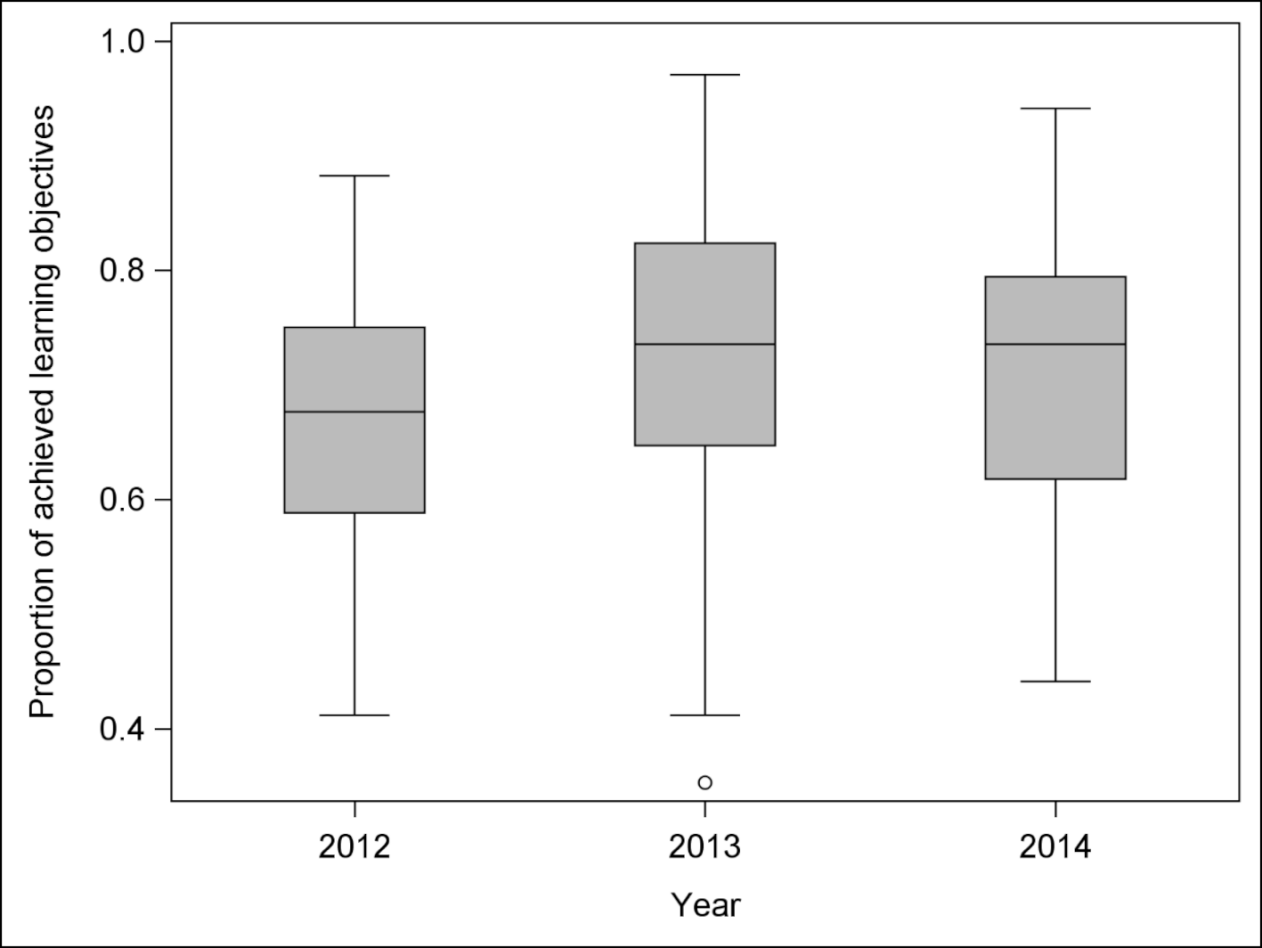
Boxplots showing distribution of the proportion of achieved LOs by student year’s cohort.

The distribution of the proportion of achieved ILOs by student year's is depicted in figure 1 (Fig. 1). The observed differences were statistically significant between the three years' cohorts (*p*=0.004). Tukey-corrected posthoc-tests indicate the following pair-wise comparisons as statistically significant: years 2012 vs. 2013 (corrected *p*=0.009) and 2012 vs. 2014 (corrected *p*=0.012). The combined analysis of both variables, academic year and academic background showed that their respective influence on the proportion of the achieved LOs was independent (the interaction of academic year and academic background was not significant, p=0.738). This means that the differences between the four academic backgrounds could be assumed equal in the three years. 

#### 3.2. Characteristics of tutors 

The distribution of PBL groups among study years and tutors' academic background is shown in table 1 (Tab. 1). The two largest groups were non-clinicians from MUV (39.4%) and near-peer students from MUV (27.8%). No significant difference regarding the distribution of academic background was observed between study years (p=0.738).

#### 3.3. ILOs achieved and additional LOs, over all PBL groups 

From the total number of all 7338 ILOs (across all 3 years, 216 PBL groups and 6 Cases) a mean of 70.8% was reached (SD 12.4% points, 95% CI: 69.2% - 72.5%). 38,3% of the groups could not achieve the aim of the faculty of at least 2/3 coverage. Furthermore, we observed 313 additional LOs outside the ILOs – with no significant difference between the 3 years (*p*=0.621).

#### 3.4. Influence of tutor's academic background on achieved ILOs and additional LOs 

**Figure 2:**
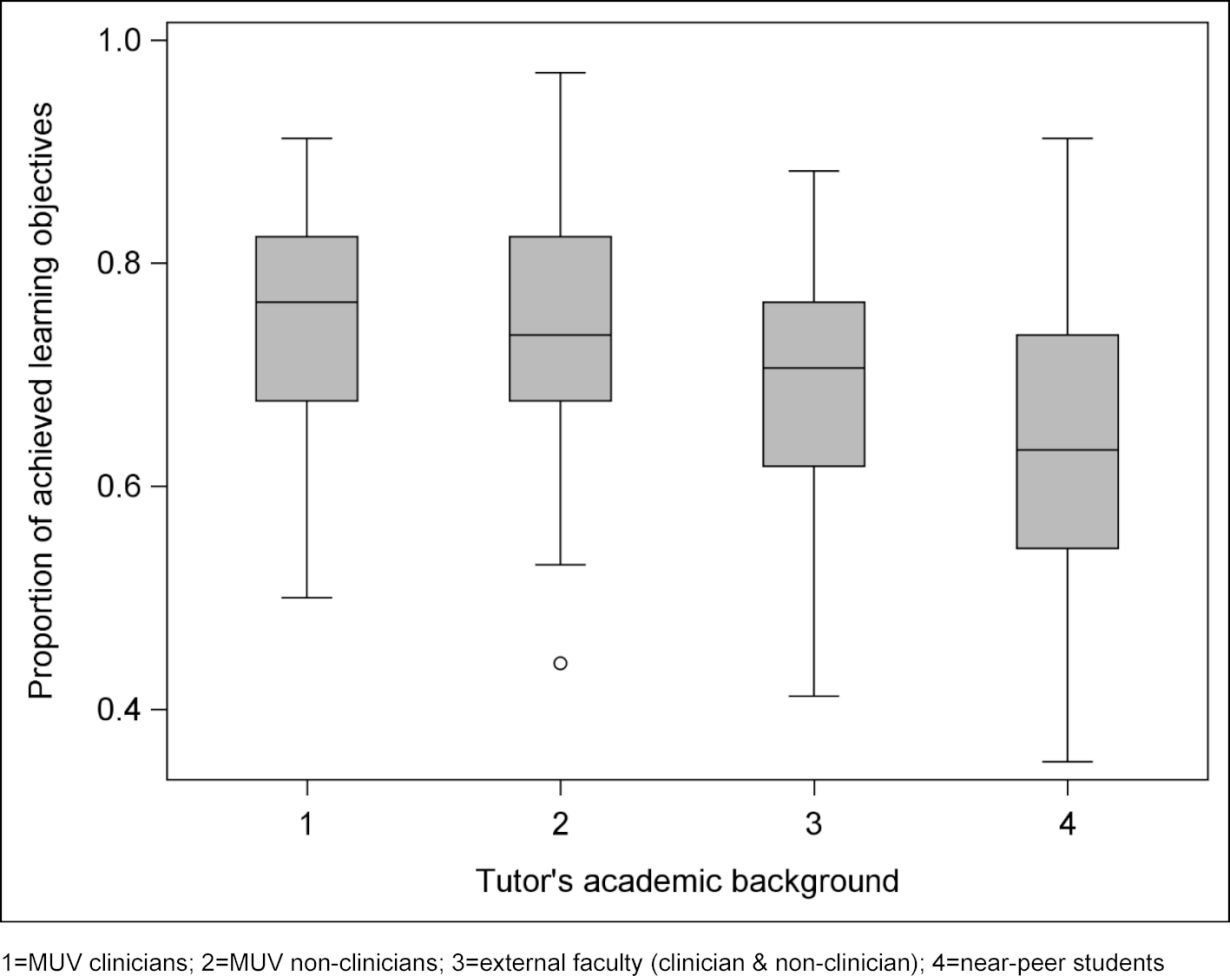
Proportion of achieved LOs across the PBL groups within each tutors’ academic background.

The proportion of achieved ILOs differed significantly in respect to the tutors' academic background (*p*<0.001) (see figure 2 (Fig. 2)). Least-squares means and 95% confidence intervals for the proportion of achieved LOs across the PBL groups within each tutor's academic background: MUV clinicians 74.3% (70.8 -77.8), MUV non-clinicians 74.2% (71.7 - 76.6), external faculty (clinicians and non-clinicians) 68.6% (64.4 - 72.8) and near-peer students 64.7% (61.8 - 67.7). The group of near-peer students differed significantly from both, MUV clinicians and MUV non-clinicians (Tukey-corrected *p*<0.001 for both comparisons). 

#### 3.5. Additional LOs 

In respect to the tutors' academic backgrounds however, we found significant differences (*p*=0.014) between the four groups with the following median percentages of additional LOs: MUV clinicians: 3.8%; MUV non-clinicians: 4.0%; external faculty (clinicians and non-clinicians): 7.4%; near-peer students: 5.1%. In case 1 of the six PBL cases, a noticeable accumulation of a certain divergent LO was stated 49 times in 216 groups (22.7%) without significant differences in respect to tutors` academic backgrounds (*p*=0.915). Furthermore, the overall percentage of additional LOs did not differ significantly between the three years. 

## 4. Discussion

The study aimed to examine whether tutor groups with different academic backgrounds achieved ILOs to a differing extent, and whether this might imply a need for further instructional efforts to elucidate potential reasons. 

We present comparative data on learning objectives worked out by all students in three years in PBL sessions guided by tutors with different academic background at a large medical university. The average proportion of 70.8% of ILOs was well within the range reported in the literature [37], [40] and the great majority of PBL groups reached the percentage of ILOs proposed by the curriculum committee. A significant increase of ILOs reached could probably only have been achieved through an unjustified external regulation or by a “dictation” of LOs which would counteract the purpose of PBL [27], [41].

The differences in the percentage of achieved LOs between the three years' cohorts were statistically significant in pair-wise comparison: years 2012 vs. 2013 and 2012 vs. 2014. With regard to the academic background of tutors there were no significant differences between the study years (*p*=0.738). We believe that making training sessions mandatory for tutors after the year 2012 was the reason for this. 

In respect to additional LOs, we found a surprisingly low proportion of 5.6% overall (which included the detection of a slightly misleading PBL case, which was replaced in subsequent years). Apparently, all tutors focussed on the ILOs and provided adequate guidance in their sessions.

The main objective of this study was to identify the impact of tutors’ academic backgrounds on the percentage of ILOs which were actually achieved. We showed significant differences between some groups and similarities between others. Also, differences were found in respect to the additional LOs, however, they were small. 

Our data confirm the finding of Regehr et al. [35] who clearly showed that content-expert and non-expert tutors achieved similar outcome measures of their students. As one alternative to MUV faculty, our PBL program included external faculty. This carried the risk of increasing heterogeneity of teaching cultures. Our data may provide an indication for this phenomenon since the mean proportion of ILOs reached was lower in this group (although not statistically significant) and the proportion of additional LOs was higher (statistically significant). 

The lowest mean proportion of ILOs (64.7%) was achieved by groups led by near-peer students. This difference was statistically significant compared to MUV faculty and deviated by 9.5 absolute percent points (which meant a relative difference of 13.7%). In addition, near-peer student-led groups showed the largest standard deviation. However, most of the groups still scored in a range acceptable for the curriculum PBL steering group and their proportion of additional LOs was lower than in the group of external faculty tutors. Near-peer student tutors are likely to have a different approach to PBL than faculty, be it conductive to the PBL process, or not. 

Mills et al. [41] have shown a non-inferior near-peer study with a satisfaction that is as high when tutored by peer-teachers compared to clinicians or non-clinical staff, but they did not mention the learning objectives.

Moust and Schmidt [42] focused their study on student's observation of student and staff tutor's behavior during PBL. Student tutors were better at understanding the nature of the problems students face in attempting to master the subject-matter. In addition, student tutors referred to end-of course examinations more frequently than staff tutors to direct student learning. In contrast, staff tutors used their subject-matter expertise more often and displayed more authoritarian behavior than student tutors. No differences were found with respect to tutor's focus on cooperation among group members. 

Tolsgaard et al. [43] stated that the quality of teaching procedural skills provided by students is comparable to or exceed that of associate professors. Despite limited clinical experience student teachers may possess substantial tacit pedagogical knowledge. 

A variety of factors contributes to this situation (see the reviews by Ross et al. [29] and Burgess al. [36]. It may be deemed beneficial that near-peer tutors are closer to the specific needs of their peers, thereby providing a more learner-friendly culture, more modern learning resources, better connection to end-of-semester assessments. This might result in a “less is more” strategy and explain the lower proportion of ILOs reached in their groups. On the less favourable side, student tutors have not yet grasped the “big picture” of medicine and are unable to relate group guidance to profound content knowledge. This could both be beneficial and/or detrimental for the group process, since content-relatedness of sessions may distract students from their extrinsic learning needs. It therefore remains open whether our finding of significantly lower proportions of ILOs reached poses a serious problem. In order to gain more insight into this issue qualitative in-depth analysis of single sessions should be performed and matched with evaluations by students and examination scores. 

Some studies already explored certain aspects of this which are of interest such as: Cianciolo et al. [44] analyzed the interactional practices during PBL tutorials and did not find differing tutor practices in respect to their professional background [42]. However, they did not link tutors' practices to achieved learning outcomes.

A special training of the near-peer student tutors may prove necessary if other quality indicators of learning showed inferior outcomes [39], [45]

### 4.1. Strenghts and Limitations

A notable strength of this study is the large sample size, which allowed for the comparison of sub-groups. In addition, it can be seen as a strength that our data were collected as a “real-life” field study rather than in an experimental setting. The replication of similar results between MUV clinicians vs. non-clinicians adds validity to our findings. 

Limitations include the mono-centred and the retrospective field study approach comprising uncontrolled influences. In addition, we did not investigate other influencing factors besides the tutors’ academic background. Further influences are likely to play a role. Also, the students` perspective is not included. Moreover, no correlation to students` exam results was made. 

It is therefore impossible at this point to gauge potential consequences of the lower proportions of ILOs reached in near-peer tutor groups. 

#### 4.2. Further research

Further research should include the students' perspective [46], [47]. A combination of evaluation questionnaires filled out by students, qualitative in-depth study of individual sessions, and exam results would help gain insight into the near-peer-led PBL group learning. 

## 5. Conclusion

In conclusion, this study yields important data on PBL tutors at a large medical university. Disregarding their academic background, the majority of tutors reached the desired range of ILOs of two thirds to three quarters.

Compared to MUV faculty, near-peer student tutors reached significantly lower proportions of ILOs. Whether and how special coaching or training would influence these results is subject to further research. In any case, our findings call for more detailed research of the tutor-group interaction in student-tutor-led PBL groups.

## Abbreviations

PBL: Problem based learning

MUV: Medical University of Vienna

LOs: learning objectives

ILOs: intended learning objectives 

## Ethics

This study was conducted according to the Helsinki Declaration guidelines and received approval by the institutional ethics committee (Ref. 1879/2015) and data protection commission of the Medical University of Vienna. 

## Acknowledgements

The authors thank all participating tutors for their PBL teaching and accurate recording and transmission of all learning objectives. 

## Competing interests

The authors declare that they have no competing interests. 

## Figures and Tables

**Table 1:**
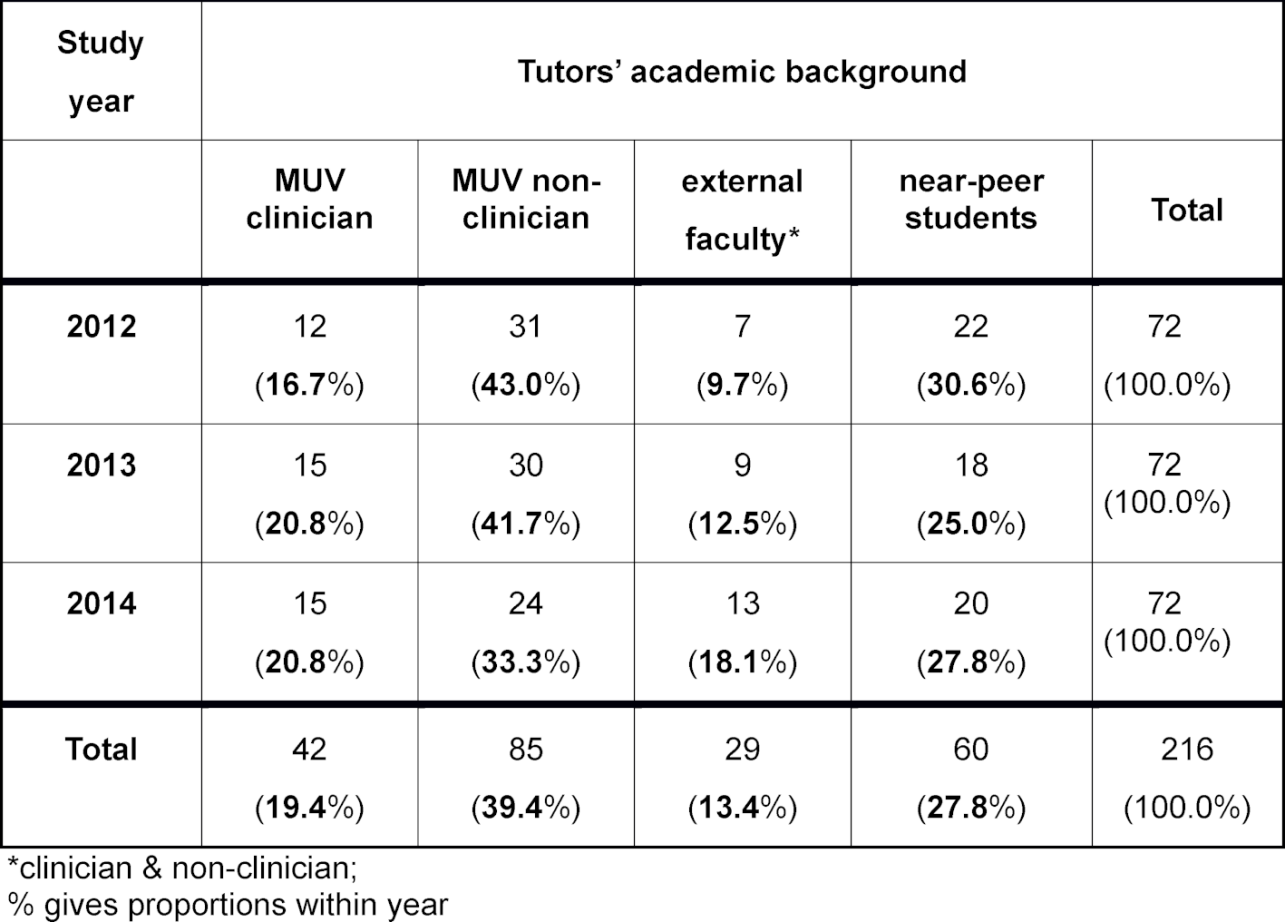
Distribution of PBL groups by tutors’ academic backgrounds and students’ years cohorts.
